# Cellulose Nanofibrils Endow Phase-Change Polyethylene Glycol with Form Control and Solid-to-gel Transition for Thermal Energy Storage

**DOI:** 10.1021/acsami.0c18623

**Published:** 2021-02-01

**Authors:** Maryam R. Yazdani, Rubina Ajdary, Ari Kankkunen, Orlando J. Rojas, Ari Seppälä

**Affiliations:** † Department of Mechanical Engineering, School of Engineering, Aalto University, Espoo 02150, Finland; ‡ Department of Bioproducts and Biosystems, School of Chemical Technology, Aalto University, Espoo 02150, Finland; § Bioproducts Institute, Departments of Chemical & Biological Engineering, Chemistry, and Wood Science, The University of British Columbia, 2360 East Mall, Vancouver BC V6T 1Z3, Canada

**Keywords:** thermal energy storage, thermal regulation, phase change material, polyethylene glycol, cellulose nanofibrils, form-stabilization, organogelation

## Abstract

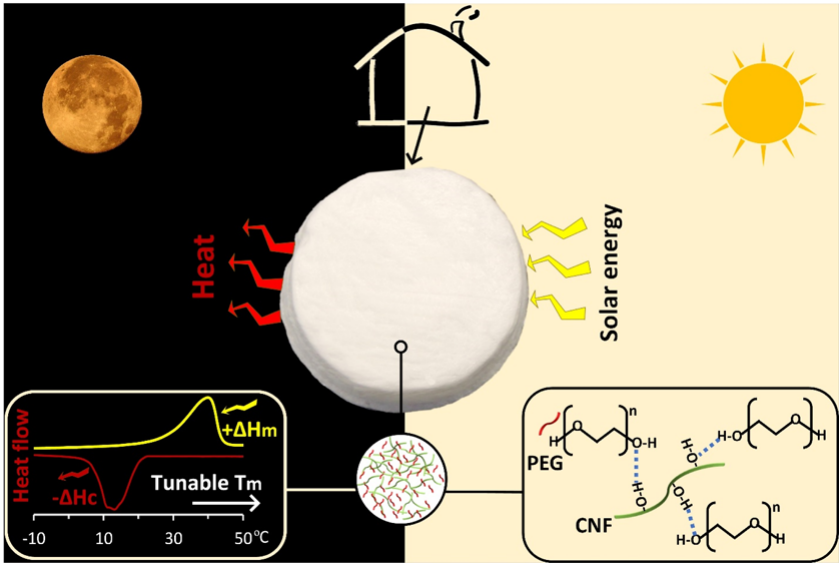

Green energy-storage materials enable the sustainable use of renewable energy and waste heat. As such, a form-stable phase-change nanohybrid (PCN) is demonstrated to solve the fluidity and leakage issues typical of phase-change materials (PCMs). Here, we introduce the advantage of solid-to-gel transition to overcome the drawbacks of typical solid-to-liquid counterparts in applications related to thermal energy storage and regulation. Polyethylene glycol (PEG) is form-stabilized with cellulose nanofibrils (CNFs) through surface interactions. The cellulosic nanofibrillar matrix is shown to act as an organogelator of highly loaded PEG melt (85 wt %) while ensuring the absence of leakage. CNFs also preserve the physical structure of the PCM and facilitate handling above its fusion temperature. The porous CNF scaffold, its crystalline structure, and the ability to hold PEG in the PCN are characterized by optical and scanning electron imaging, infrared spectroscopy, and X-ray diffraction. By the selection of the PEG molecular mass, the lightweight PCN provides a tailorable fusion temperature in the range between 18 and 65 °C for a latent heat storage of up to 146 J/g. The proposed PCN shows remarkable repeatability in latent heat storage after 100 heating/cooling cycles as assessed by differential scanning calorimetry. The thermal regulation and light-to-heat conversion of the PCN are confirmed via infrared thermal imaging under simulated sunlight and in a thermal chamber, outperforming those of a reference, commercial insulation material. Our PCN is easily processed as a structurally stable design, including three-dimensional, two-dimensional (films), and one-dimensional (filaments) materials; they are, respectively, synthesized by direct ink writing, casting/molding, and wet spinning. We demonstrate the prospects of the lightweight, green nanohybrid for smart-energy buildings and waste heat-generating electronics for thermal energy storage and management.

## 1 Introduction

Decarbonization of the energy sector is a major challenge in this century, centered around the environment.^1^ Devising green alternatives for the use of renewable energy and industrial waste heat can pave the way to a carbon-neutral future. Because of their intermittent nature, however, the capture of such sustainable energy resources relies on energy-storage technologies. Energy management can bridge supply and demand by storing excess energy, mainly in the form of heat or electricity. During the past few decades, thermal energy storage (TES) based on sensible and latent heats of thermal materials has attracted growing interest.^2,3^ In this context, phase-change materials (PCMs) enable the storage and recovery of thermal energy through the enthalpy of phase transition, mainly from solid-to-liquid and liquid-to-solid at a nearly constant temperature. PCMs can thus offer effective capture and regulation of high energy densities across a wide temperature range, favoring many temperature-dependent applications.^4−6^ For instance, PCMs possessing fusion temperatures relevant to the human thermal comfort are attractive for regulation in buildings, furniture, and wearables.^7^ Paraffins and fatty acids have been proposed in buildings, for example, for use in the construction walls or in storage units, to harvest solar energy and moderate the energy consumption.^8−10^ Thus, incorporation of PCMs in buildings improves their energy efficiency and maximizes renewable energy in the construction sector, which is responsible for a large share of the global energy demand. Various other segments of industry, such as active-packaging, refrigeration, electronics, sensors, and those associated with waste heat recovery can benefit from these versatile materials.^11,12^ For example, PCM-activated fabrics are used as a thermal buffer in response to drastic environmental temperature changes, for example, to maintain the body comfort, which is attractive for industrial, medical, and aerospace purposes.^13^ Lithium-ion batteries protected with PCMs have been shown to be more resistant against breakdown in cold temperatures.^14^ Likewise, PCM-based designs can be used for heat capture and thermal protection of portable and transient electronic devices,^15^ such as computers and 5G poles that generate considerable amounts of heat. The major challenges in the actual use of PCMs include leakage and associated volume changes in the liquid state, need for special liquid containers, incompatibility with support materials, and instability, all of which lead to high operation expenditures.^11,16^

Confinement, encapsulation, and form-stabilization of PCMs with polymers,^16−19^ inorganics,^20−22^ and porous materials^23,24^ are among the methods considered in solving the leakage of PCMs and enhancing their thermochemical durability. Encapsulation is usually used to create a shell-like structure around small droplets of hydrophobic PCMs, such as paraffins and fatty acids.^7,25,26^ Confinement has been achieved mainly by using porous silica, carbons, metal frameworks, and wood supports.^16,24,27,28^ Form-stabilization^3^ results from the affinity of PCM to the support matrices through blending and adsorption. Hence, form-stabilization is obtained, for instance, by exploiting the miscibility between the PCM and the support matrix through compatible intermolecular interactions. Stabilized fluidity enables easy handling and wide applicability by preventing leakage above the melting point of the PCM. However, most of the PCMs supported by given matrices have shown low latent heat storage and poor biocompatibility, limiting their applications.^29^

Polyethylene glycol (PEG) provides favorable intrinsic characteristics such as biocompatibility, nontoxicity, high resistance to corrosion, high latent heat of fusion, and tunable fusion temperature (by selection of molecular mass), as well as a suitable cost structure.^11,22,23^ However, like many other solid-to-liquid PCMs, PEG suffers from leakage and volume change in the melt state, limiting its application. Herein, we propose cellulose nanofibrils (CNFs), originating from ubiquitous renewable resources such as wood, plants, and microorganisms, to act as biocompatible lightweight support for PEG through organogelation. As a class of soft materials, organogels can capture large amounts of organic solvents. CNFs are widely utilized as a physical reinforcement in emerging bioproducts within a variety of fields; however, CNF consideration for thermal energy enhancement is still quite rare.^30−33^ Emulsification with CNFs, for example, has been proposed for encapsulation of oil-based paraffin.^25^ CNFs can be obtained through mechanical and/or chemical processing of wood pulp in the form of aqueous suspended colloidal nanofibrils.^34^ CNFs provide high functionality owing to the high surface area, high aspect ratio (4 nm < width < 20 nm and 500 nm < length < 2000 nm),^34^ and surface functional (hydroxyl and carboxyl) groups, which can promote organogelation of melted PEG in form-stabilization. Furthermore, tunable physical properties of CNFs enable easy processing and structuring into materials, including those in three-dimensional (3D), two-dimensional (2D), and one-dimensional (1D) forms. The use of CNFs to enhance PCMs is expected to increase the environmental sustainability of energy-storage technologies.

**Scheme 1:**
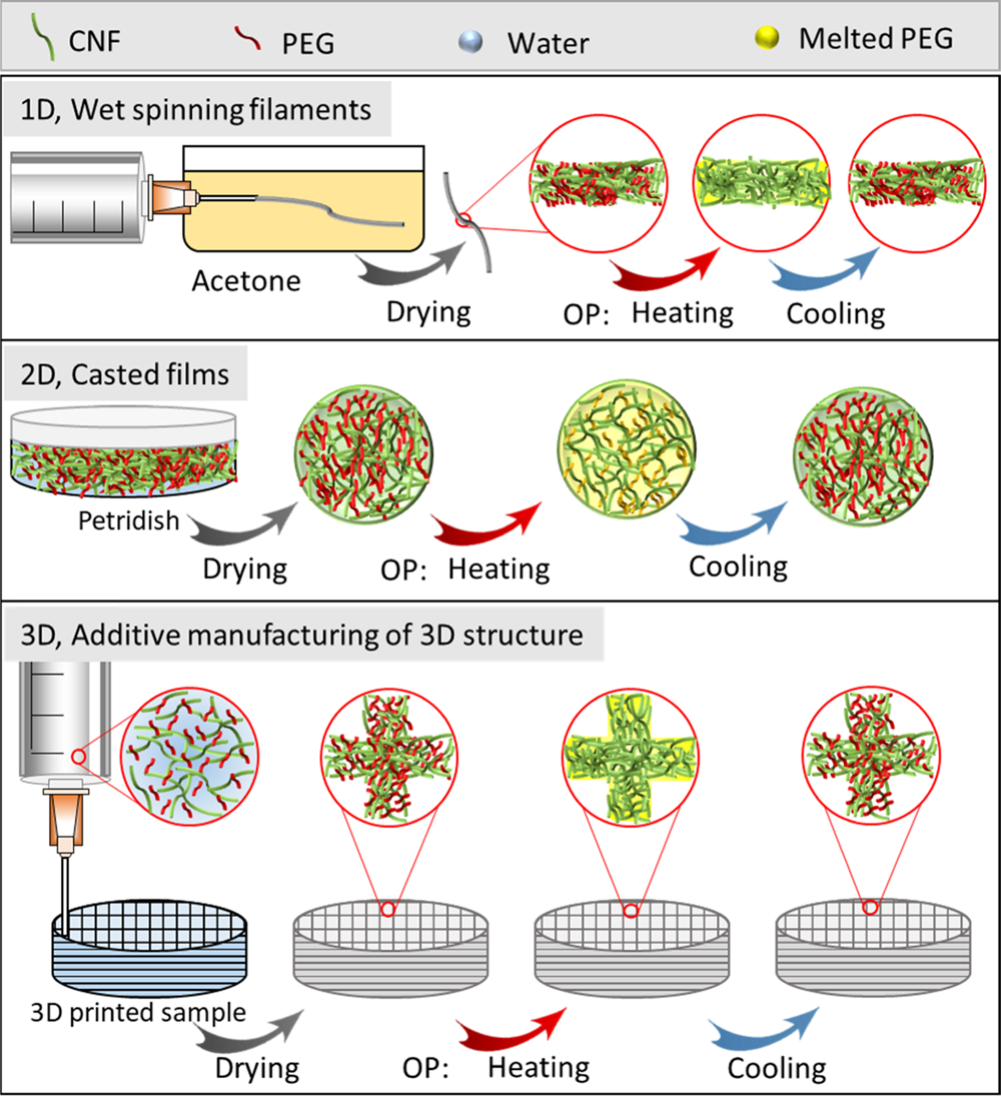
Schematic Illustration of PEG-CNF Hydrogel Processing into 1D, 2D, and 3D Structures Through Wet Spinning, Solution Casting, and Additive Manufacturing The principle of operation in energy storage is shown by melting through heating and crystallization via cooling.

To this end, we demonstrate a leakage-proof phase-change nanohybrid, herein referred to as PCN, prepared through a simple aqueous blending process that combines PEG with CNF matrices. The blend is easily processed via additive manufacturing, casting/molding, and wet spinning to fabricate controlled nanostructures in 3D, 2D, and 1D shapes (Scheme 1). The nanohybrid is formed by the hybridization of nanocellulose, as the nanoscale additive, with PCM, as the main component, through secondary intermolecular forces. Owing to its nanosize, nanocellulose creates a bulky scaffold of large surface area that stably holds the PCM in the melt state and enables different processing methods. CNFs make possible a high PEG loading because of their affinity. The PEG phase can capture and release heat in response to temperature variations through a solid-to-gel transformation rather than a typical solid-to-liquid transition. Most importantly, PEG provides a tailorable fusion temperature by selection of molecular mass, for example, from ∼20 °C to 65 °C, using an average molecular mass (*M*_n_) between 600 and 8000 g/mol. This enables tailoring the operation temperature as a function of PEG’s molecular weight, significantly opening applications that require a specific constant working temperature.^3^ The molecular interactions, for example, hydrogen and van der Waals bonding, between the PEG melt and the high surface area created by the CNF network, support a high loading level and leakage-proof characteristics above the fusion temperature. The proposed lightweight, form-stable PCN can be utilized in energy storage and thermal management for buildings, electronics, and active packaging, among others. Bio-based PCM incorporated in building walls,^10^ for example, can potentially provide a comfortable indoor climate (temperature and humidity) for dwellers.

## 2 Materials and Methods

### 2.1 Materials

PEG samples (Sigma-Aldrich) of a given molecular mass (*M*_n_, g/mol) were selected for tuning the fusion temperature, namely, PEG600 (*M*_n_ 600, fusion at 17–22 °C), PEG1000 (*M*_n_ 950–1050, fusion at 37 °C), PEG4000 (*M*_n_ 4000, fusion at 48–55 °C), PEG6000 (*M*_n_ 6000, fusion at 58–60 °C), and PEG8000 (*M*_n_ 8000, fusion 58–60 °C). The chemicals used for the CNF preparation, including NaOH, NaClO, and 2,2,6,6-tetramethylpiperidine-1-oxyl (TEMPO), were all purchased from Sigma-Aldrich.

### 2.2 CNF Preparation

Never-dried Birch cellulose pulp was used to prepare CNFs through TEMPO-mediated oxidation. TEMPO (0.013 mmol/g) and sodium bromide (0.13 mmol/g) were mixed with the aqueous suspension of cellulose pulp (17.03 wt %). Sodium hypochlorite (NaClO) (5 mmol/g) was then added to the system. NaOH (0.1 M) was used to maintain the suspension pH at 10. After 6 h of continuous stirring, the CNF was rinsed with distilled water and disintegrated by a microfluidizer (M-110P, Microfluidics In., Newton, MA, USA). The final CNF suspension was dehydrated to 1.7 wt % at room temperature under continuous stirring and stored in cold for further usage.

### 2.3 Preparation of PCN

The PCN was prepared through a facile and low-energy procedure that used no other solvent but water. Different PEG-CNF hydrogel compositions were prepared by adding respective amounts of PEG into the CNF suspension. First, the given PEG was dissolved in distilled water (mass of PEG/mass of water ≈1) at elevated temperatures, preferably below the fusion temperature, for example, 30 °C for PEG1000 and 50 °C for PEG4000-8000. In the case of PEG600, the solution was prepared at room temperature. The dissolved PEG solution was then added to the CNF suspension (1.7 wt %) for a dry mass composition of PEG/CNF of either 75:25, 80:20, or 85:15 wt %. The compositions were mixed for 2 h to obtain a homogeneous hydrogel. Rheological analyses were performed with the PEG-CNF hydrogels under the processing pressure (see the Supporting Information), given that the rheological response determines the final structure of the nanohybrid. The prepared hydrogels were then processed through 3D printing, solution casting/molding, and wet spinning (Scheme 1). An example of a 3D-printed PEG-CNF hydrogel is presented in Figure S1. The hydrogel compositions were dried using the given condition (Table 1) and stored for further characterization and thermal energy measurements.

**Table 1:** Latent Heat Storage of PEG and PCN Compositions Measured with DSC at a 5 K/min Scan Rate, Tuning the Thermal Properties by Molecular Mass and Weight Percentage of PEG in the PCN^a^

composition	PEG (*M*_n_)	CNF	*T*_m_ (°C)	Δ*H*_m_ (J g^–1^)	Δ*H*_c_ (J g^–1^)	*C*_P,s_ (J g^–1^ K^–1^)	C_P,l_(J g^–1^ K^–1^)
	PEG 8000		67.2	196.0	-187.7	1.36	1.88
A	85%	15%	64.6	137.0	–133.0	1.36	1.98
A1	80%	20%	61.2	137.7	–136.0	1.50	2.12^b^
A2	75%	25%	62.2	99.6	–98.0	1.36	1.90
	PEG 6000		67.2	192.0	–182	1.35	2.04
B	85%	15%	65.4	146.2	–139.0	1.41	2.01
B1	80%	20%	62.8	129.0	–124.5	1.46	1.88
B2	75%	25%	59.4	95.1	–93.3	1.35	1.83
	PEG 4000		65.4	182.0	–177.0	1.24	1.93
C	85%	15%	62.4	140.0	−137.2	1.76	2.25
C1	80%	20%	62.5	137.3	–131.0	1.42	2.09
C2	75%	25%	60.4	120.2	–118.9	1.44	1.94
	PEG 1000		42.2	164.2	–157.3	1.57	2.09
D	85%	15%	39.0	113.0	–108.0	2.08	2.00
D1	80%	20%	40.0	118.6	–112.0	1.74^b^	2.2
D2	75%	25%	40.0	95.2	–90.6	1.74	2.03
	PEG 600		24.4	132.0	–125.1	1.38	1.91
E	85%	15%	21.4	99.0	–90.0	1.66	2.05
E1	80%	20%	22.4	92.0	–82.0	2.16	2.47
E2	75%	25%	17.8	81.3	–71.0	1.63	1.94

a *T*_m_ is the peak temperature of fusion; Δ*H*_m_ is the latent heat of melting; Δ*H*_c_ is the latent heat of crystallization. The latent heat is determined as the area under the DSC peaks. *C*_P,s_ and *C*_P,l_ are the specific heat capacities of the solid and liquid forms. *C*_p_ values were determined on heating at 75 °C for the liquid and at 10 °C for the solid. In the case of PEG600, *C*_p,l_ values are for 50 °C and *C*_p,s_ values are for −15 °C. See Figures 6 and 7 for the corresponding DSC and *C*_p_ profiles.

b Of note, *C*_p,1_ of A1 is for 77 °C and *C*_p,s_ of D1 is for 5 °C.

**Figure 1:**
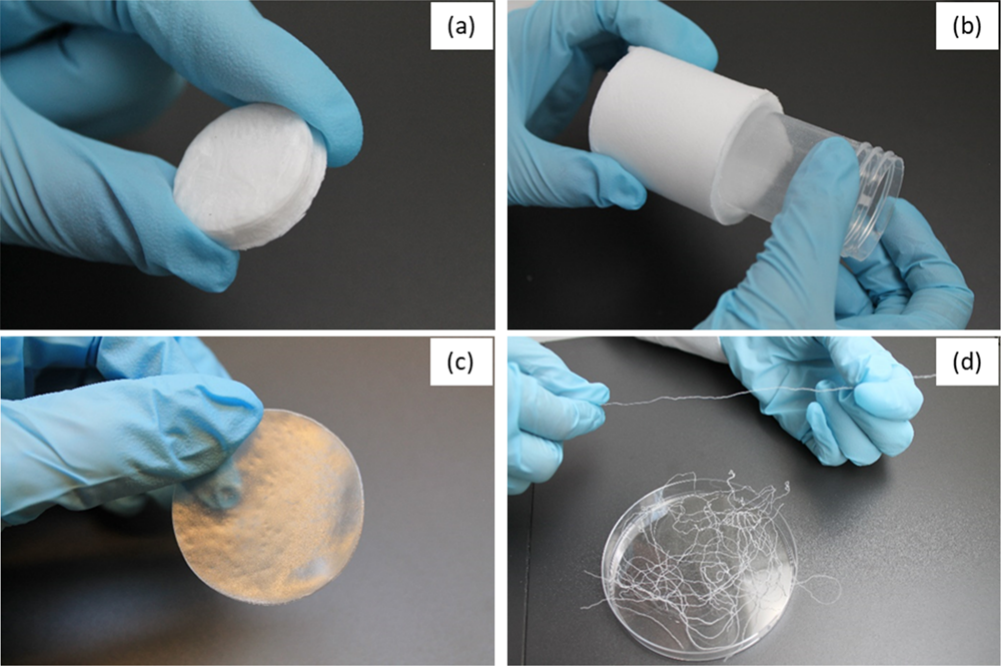
PCN samples formed in 3D printed, 2D film, and 1D filament forms (C2 in Table 1) and produced by (a) additive manufacturing, (b,c) casting/molding, and (d) wet spinning. They demonstrate excellent structural stability.

The 3D structured samples were fabricated with a BIOX bioprinter (CELLINK, Sweden) with pneumatic print heads. The system used pneumatic 3 mL syringes and a blunt 20G needle. The diameter of the needle was 0.63 mm, and the 3D print surface used a plastic Petri dish. The 3D-printed sample consisted of a pellet with a diameter of 27 mm and a height of 5 mm produced by the rectilinear infill pattern, Figure S1a. After printing, the samples were first frozen overnight and dried under vacuum for 48 h, Figure 1a. The filaments were produced through wet spinning. The PEG-CNF hydrogel was pumped into a cold acetone coagulation bath using a Nexus 6000 High Force High Pressure Syringe Pump (Chemyx) equipped with a 1.2 × 40 mm blunt needle (B. Braun Melsungen AG, Germany). The film samples were prepared by casting in a plastic Petri dish at room temperature. The dried samples, 3D object, 2D film, and 1D filament (Figure 1), were stored at room temperature for further analyses.

### 2.4 Characterization

Scanning electron microscopy (SEM) imaging was performed to observe the structural morphology. SEM was carried out on a Zeiss Sigma VP microscope (Germany) at 2 kV accelerated voltage and under vacuum condition. Prior to microscopy, a sputter coater (LECIA EM ACE600) was used to coat the samples with a thin layer of gold palladium alloy (4 nm). The samples were also observed with a Leica DM4500 optical microscope equipped with a Linkam TMS91 heating–cooling stage. Attenuated total reflection Fourier transform infrared spectroscopy (ATR-FTIR) was done on a Spectrum Two PerkinElmer spectrometer in the 4000–500 cm^–1^ range at 32 cm^–1^ resolution using 12 scans. A Rigaku SmartLab X-ray diffractometer was used to perform X-ray diffraction (XRD) using a rotating anode X-ray source (9 kW, Cu Kα_1_) and a HyPix-3000 2D detector. The dynamic mechanical properties of the PCN were studied using dynamic mechanical analysis (DMA, TA Instruments Q800) operated in the tensile mode. The program was set at a constant frequency of 1 Hz within 30 to 100 °C range under a 5 °C/min heating rate. The stress–strain tests were performed with the DMA at 30 °C under a ramp loading rate of 1 N/min up to 18 N. The sample dimensions were approximately 0.08 × 5.4 × 20 mm (thickness × width × length). Four replicates were examined.

### 2.5 Thermal Energy Measurements

Differential scanning calorimetry (DSC) was employed to measure the fusion temperature and latent heat (enthalpy) using a Netzsch DSC204F1 Phoenix DSC instrument. A sample and an alumina reference were exposed to a heating–cooling program, and the difference of the heat flow rate as a function of temperature was recorded as the DSC curve. The enthalpies of crystallization (Δ*H*_c_) and melting (Δ*H*_m_) reactions were determined as the area under the exothermic and endothermic DSC peaks, respectively. The fusion temperature (*T*_m_) was assigned to the peak temperature. The DSC program included a dynamic temperature range, within −20 to 80 °C, consisting of four consecutive cycles at a 5 K/min scan rate. Given their low fusion temperature, the compositions, including PEG600, were subjected to the dynamic DSC program in the temperature range between −40 and 60 °C. Three replicates were carried out for each composition. To test the repeatability of the phase-change behavior, 100 consecutive DSC heating-cooling cycles were performed with the PCN (5 K/min scan rate). The specific heat capacity (*C*_p_) was measured by DSC according to the Sapphire correction *C*_p_ method. The DSC sample was prepared by placing 10–20 mg of the material within a standard aluminum crucible and lid. Thermogravimetric analyses (TGAs) were performed on a Netzsch STA 449 Jupiter instrument from 30 to 450 °C under 10 K/min ramp heating and N_2_ condition.

Temperature distribution images were taken by an infrared thermal camera (FLER SC7000). The samples were placed on a silicon support under two light sources located on the sides at 40 cm distance and 45° angle, which distributed the light evenly on the sample, Figure S2a. The light sources were used to simulate solar energy for heating and consecutive cooling through a manual switch. The samples included the 3D-printed PCN (27 mm diameter × 5 mm height and 0.17 g mass) as well as pure PEG and CNF of similar dimensions (2.1 and 0.06 g, respectively). The measurements in the thermal chamber were performed in a temperature-controlled system programed to increase the temperature from room values to 70 °C. The measurement was carried out with a bottle filled with water and covered with a 10 mm PCN layer (Figure S2b). The results were compared with those using a commercial Armaflex insulation material (Armacell, thermal conductivity of 0.040 W m^–1^ K^–1^ according to the product data sheet and 10 mm thickness) as well as a bare bottle. Note that Armaflex is a commercial elastomeric thermal insulation material commonly used to prevent heat gain in refrigeration and air-conditioning systems as well as heat losses in hot water plumbing and heating systems relevant to industrial and solar applications. The thermal conductivity of the PCN and CNF samples was measured using the modified transient source plane method on a C-Therm thermal conductivity analyzer (C-Therm TCi). The 3D-printed samples of pure CNF and PCN (C2) were examined at room temperature using three replicates (Figures 1a and S3c).

## 3 Results and Discussion

### 3.1 Physicochemical Properties of PCN

Figures 1 and S3 show the PCN and CNF systems developed through different processing methods. The PCN can be synthesized as 3D objects, 2D films (sheets), and 1D filaments, providing excellent structural stability and lightweight. Such systems are expected to enable a wide range of energy storage and regulation applications, including buildings and heat-generating electronics.

**Figure 2:**
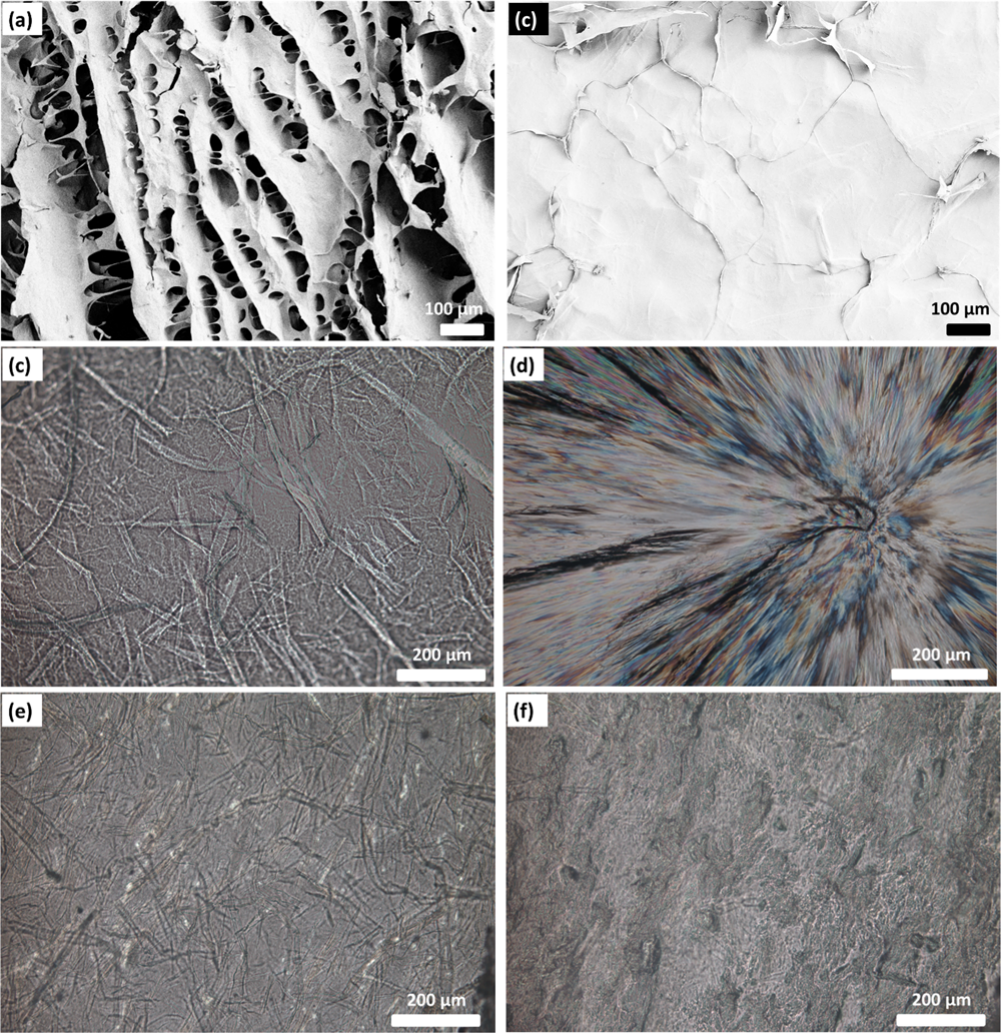
SEM images of PCN (C2 in Table 1). (a) CNF and (b) PCN (3D-printed samples); see Figure S4 for the SEM images of the filament and the film. POM images of (c) CNF, (d) PEG, and (e) PCN in the melt state and (f) PCN in the solid state.

**Figure 3:**
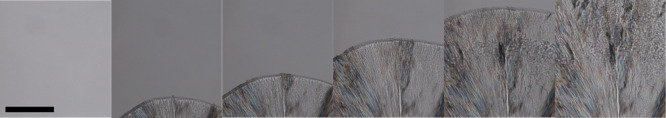
Spherulite formation upon the PEG melt (far left image) cooling at 5 K/min from 70 °C. The size of scale bar is 200 μm.

The mass of 3D-printed cylindrical PCN (Figure 1a) was 0.17 g, while that of pure PEG with similar dimensions was 2.1 g. This indicates a ∼92% weight reduction in the PCN. This lightweight feature is beneficial in applications such as those in the electronics, textiles, and aerospace sectors;^35^ it also enables easy/simple handling, installation, and transportation. Thanks to their low weight, such materials produce low CO_2_ emissions during transport.^36^ The morphological structure of the PCN was observed by SEM (Figures 2 and S4). SEM images of the PCN in 3D printed, film, and filament forms were compared with those of neat CNF. It is observed that the CNF creates a porous scaffold that can be utilized to hold the PCM. The PCN possesses the 3D fibrillar network comprising CNF filled with thermally active PEG. A similar filling morphology was previously observed for PEG in aerogels.^37,38^ SEM confirms the homogeneous distribution of PEG within CNF matrices with no phase segregation of the components, originating from their compatibility, leading to a high PEG loading (85%) in the system without leakage in the melt state. Polarized optical microscopy (POM) showed the crystallization morphology during the cooling process, Figures 2 and 3. POM reveals a fibrillar network morphology of CNF and PEG spherulitic crystalline structure at room temperature. Spherulite formation upon cooling is illustrated in Figure 3. The Supporting Information includes a video of pure PEG with spherulite formation upon crystallization from the melt state. Spherulites are crystalline lamellae with 3D superstructures, the most common morphology for the crystallization process of polymers from the melt. Spherulite formation generally occurs in three stages of nuclei formation, crystal growth, and secondary crystallization involving an increment in the crystallinity and thickness of lamellar crystals.^39^Figure 2e,f shows the PCN at the melt (70 °C) and crystalline (30 °C) states, respectively. In the melt state, the cellulosic nanofibrillar network is clearly observed, which is covered with the PEG spherulites after crystallization. Optical microscopy confirms the retention of PEG melt in the nanocellulosic matrix (see also the video of PCN crystallization on cooling in the Supporting Information). The video visualizes the formation of PEG spherulites within CNF matrices. The addition of the CNF phase affects the properties of the PEG, including the overall crystallinity, the crystal morphology, the spherulite dimension, and the amorphous–crystal interface. A similar spherulitic crystallization of PEG within biopolymeric matrices was observed elsewhere.^29,40,41^

**Figure 4:**
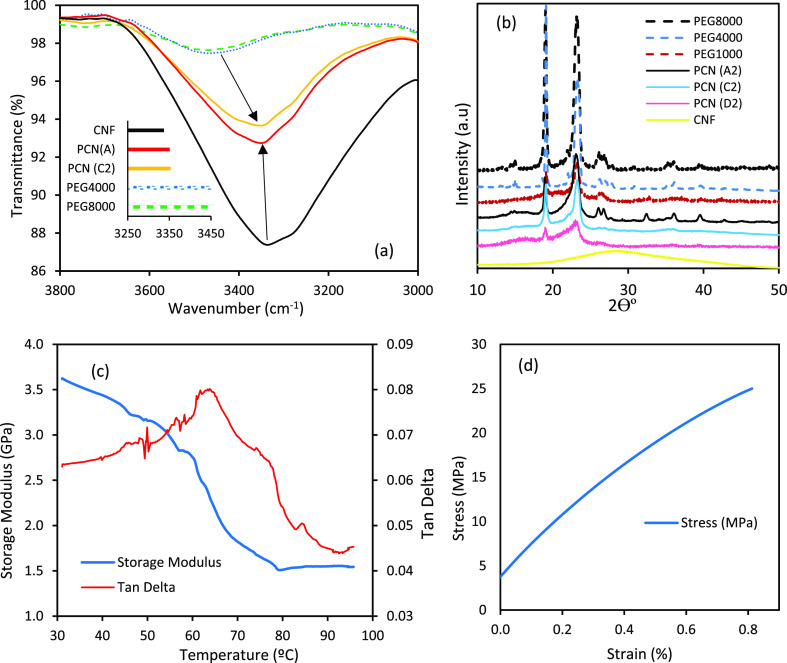
(a) ATR-FTIR spectra of PEG4000 and PEG8000 as well as PCN (C2 and A) and CNF at the −O–H stretching region at the wavenumber of the maximum hydroxyl vibrational band. (b) XRD profiles of PEG (*M*_n_ 8000, 4000, and 1000), CNF, and PCN (A2, C2, and D2). (c) Storage modulus and tan delta profiles of a PCN film (C2) measured using DMA and (d) the stress–strain profile of the PCN film (C2).

FTIR spectroscopy was used to reveal the intermolecular interactions between the components coupled in the PCN. Figures 4a and S1d show the FTIR spectra of the PCN and the starting materials. The spectra of the PCN results from a combination of those of CNF and PEG with no new chemical bonding, confirming the nature of the interactions between the constituents, which was physical. The broad peak at 3600–3100 cm^–1^ is attributed to the stretching vibration of O–H groups. Hydrogen bonding affects the infrared spectrum of materials by broadening the characteristic −O–H stretching band at decreased frequencies for the associated molecules. The hydroxyl vibrational band indicates 120 cm^–1^ shift in the PCN spectrum compared to that of PEG (Figure 4a), suggesting intermolecular hydrogen bonding.^3^

XRD was performed to study the crystallinity of the PCN and the starting materials. Figure 4b presents the XRD patterns of PEG (*M*_n_ 1000, 4000, and 8000), PCN (A2, C2, and D2), and CNF films. The CNF shows a single broad peak at 2θ = 28°, suggesting some level of molecular orientation and hydrogen bonding between the CNFs.^3^ PEG shows two well-defined peaks at 2θ = 19.1° (the highest intensity) and 23.3° related to the (120) and (032) lattice planes of the PEG crystal, respectively.^37,42^ The two characteristic peaks of PEG are observed in an amorphous background (XRD profile of PCN), which confirms the presence of both crystalline PEG and amorphous CNF. Figure 4b indicates that the intensity of the diffraction pattern increases with the molecular size, suggesting that a higher molecular mass of PEG provides lower segmental mobility and easier geometrical alignment, forming crystalline lamellae compared to those formed from PEG of lower molecular mass.^42^ Furthermore, the crystal thickness of PEG increases by increasing the average molecular mass, which is consistent with the higher enthalpy of crystallization of PEG of higher molecular mass (Table 1), as discussed in the following sections.

The dynamic mechanical property of PCN is illustrated in Figure 4c including the storage modulus and tan delta in temperature curves. The storage modulus remains at ∼3–3.5 GPa within 30–50 °C, corresponding to the solid state. A modulus drop is observed in the temperature range from 50 to 80 °C, related to the solid–liquid transition. After the phase transition, a “plateau” at 1.5 GPa is observed, suggesting reinforcement by hydrogen-bonding interactions within the cellulosic nanofibrillar network. A similar entangling behavior was reported previously for CNF-reinforced polylactic acid.^43^ The PCN film presented a tensile strength of 28 MPa and strain at a failure of 1% (Figure 4d). Pure PEG was too weak for measurement. The improvement in the mechanical properties, especially in the strength of the nanohybrid, is due to the insertion of the PCM within CNF matrices. The load-bearing properties are beneficial in the application of such bioproducts. Load-bearing structures with lightweight materials, for example, are used as alternatives for concrete or masonry structures used in new buildings.^36^ Renewable building materials, with low embodied energy and emissions, enhance the life cycle of constructions. Furthermore, owing to their low weight, lightweight materials cause lower greenhouse gas emissions during transport. Lightweight thermal protection materials for aerospace activities also require tensile and mechanical strengths to prevent deflection and deformation under harsh environments.^35^

### 3.2 PCN Fluid Retention

**Figure 5:**
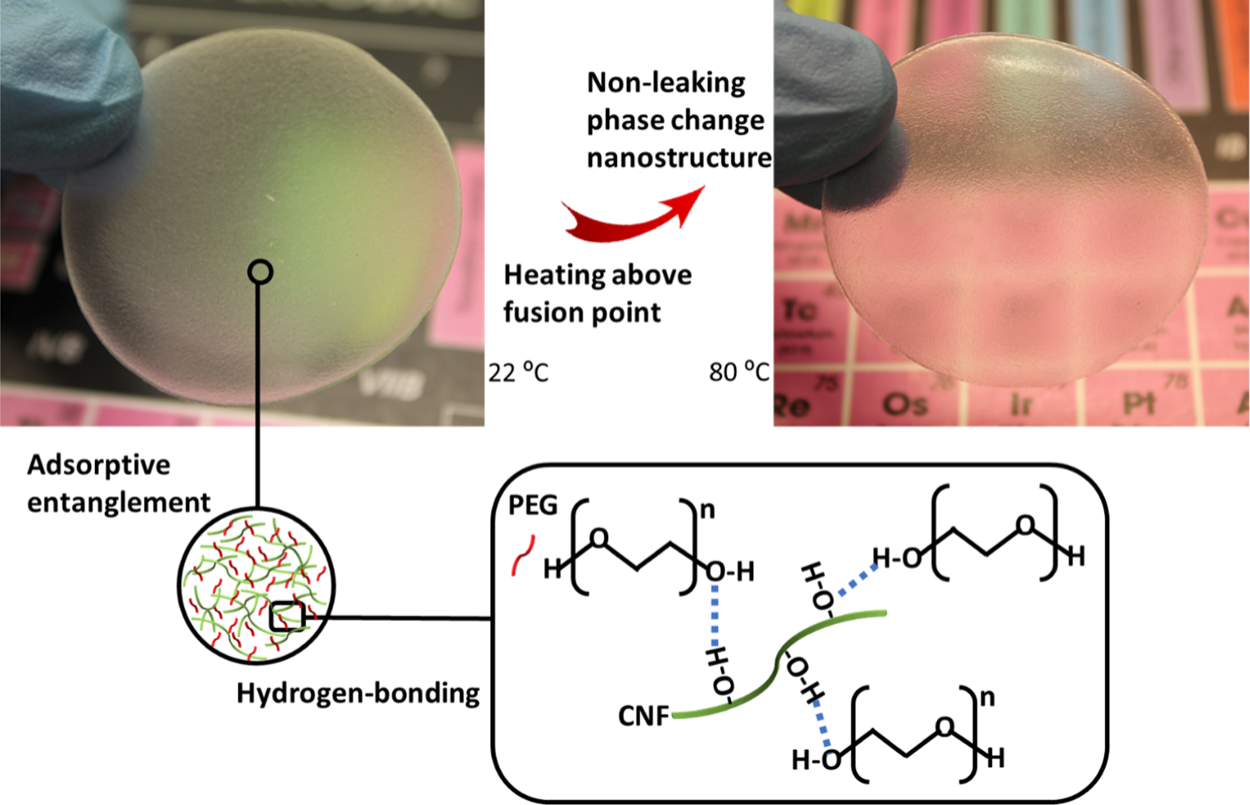
Form-stability and leakage prevention of PCN films developed from PEG and CNF through entanglement and hydrogen bonding. The PCN film was exposed to heat at 80 °C for 2 h. See Figure S5 in relation to the form-stability of 3D-printed samples.

The nanoscale dimensions of nanocellulose enables the creation of highly porous structures with an ultralow density (Figure 2a). Cellulose structures display both hydrophilic and hydrophobic planes. The amphiphilicity of CNFs provides adsorption capacity for both polar and nonpolar liquids.^44^ Therefore, CNFs can be used to retain fluids, that is, enhance the form-stability for the liquid PCMs, even in very thin structures (Figure 5 for PCN films). The form-stability of 3D-printed PCN and fluidity of bulk PEG exposed to heat at 80 °C are compared in Figure S5. PEG melting causes a large volume change, leakage, and handling issues. On the other hand, PCN provides form-stable structures at the melt state, preventing the leakage of the PCM melt by the CNF network, owing to the high miscibility and physical interactions (Figure 5) that lead to a solid-to-gel transformation, rather than a typical solid-to-liquid transition. The PCN can be classified as an organogel^45^ that captures large amounts of melted PEG as the organic phase. Polymer gelation can be exploited to form-stabilize PCMs above their fusion point. In such cases, the polymer is often dissolved in an organic liquid paraffin^46^ or inorganic salt hydrates.^47,48^ The physical gelation occurs when macromolecular networks are created through secondary interactions, for example, electrostatic, hydrogen bonds, and van der Waals. Thus, CNF matrices act as organogelators for the PEG melt and capture a considerable amount of PEG while holding the physical structure and easing the handling of the PCM above the fusion temperature. The physical interactions between PEG and CNFs are demonstrated (FTIR in Figures 4a and S1d as well as the DSC analyses in the following section) and schematically illustrated in Figure 5.

### 3.3 PCN TES

The latent heat storage of the developed nanohybrid, that is, the phase-change enthalpies and temperatures, depends on the PCM/CNF ratio and PEG’s molecular weight. Table 1 includes a compilation of the phase-change properties of PCN of varying compositions, including PEG/CNF ratios and the molecular weight. As seen in Table 1, increasing the PEG content in the PCN generally results in increased energy stored during melting (Δ*H*_m_) and released during crystallization (Δ*H*_c_). In this study, we examined PEG/CNF ratios of 85:15, 80:20, and 75:25 wt %, yet higher loading of PEG can still be explored in the future to maximize the loading. The latent heat depended on the molecular mass of PEG; larger *M*_n_ generally results in higher fusion enthalpy. The stored heat or enthalpy through melting ranges between 81 and 146 J/g; meanwhile, the released heat through crystallization enthalpy varies between −71 and–142 J/g. The small differences between the melting and crystallization enthalpies are due to the difference in the specific heat of the melt and solid states
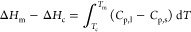
1where *C*_p,l_ and *C*_p,s_ are the specific heat capacities of the liquid and solid, respectively, and *T* is the temperature. The molecular size of PEG has a major effect on the fusion temperature, that is, the operation temperature of the storage material. The fusion temperature of PEG increases from 24.4 to 65.4 °C by increasing the molecular mass from 600 to 4000 g/mol, while a further increase of molecular mass to 8000 g/mol leads to smaller changes in the fusion temperature, 67.2 °C (see Table 1). A tailorable operation temperature favors different applications fitting specific requirements; for example, hot water supply requires higher working temperatures (40–80 °C)^6,49,50^ compared with that of indoor temperature-regulation in buildings (19–25 °C).^49,51^ An increased CNF content, from 15 to 25 wt % in the PCN, generally results in a reduction of fusion temperature. For instance, the *T*_m_ values for PCN prepared with PEG4000 (C-compositions) are reduced from 62.4 to 57.4 °C. The enthalpy of fusion of the PCN compositions (e.g., 146 J/g) competes with those of existing PCMs, such as paraffins (100–200 J/g)^8^ and recently reported PCM composites, including encapsulated fatty acids in polystyrene hollow fibers (147 J/g),^12^ encapsulated paraffin in nanocellulose (139 J/g),^25^ electrospun fiber-supported PCMs (∼166 J/g),^52^ electrospun fibers of PEG-cellulose acetate (120 J/g),^18^ and PEG incorporated in isocyanate-terminated prepolymer and tetrahydroxy prepolymer (98 J/g).^53^ Note that most existing PCMs suffer from drawbacks such as volume change, leakage, fossil fuel-based origin, and instability. By contrast, the PCN developed herein provides several advantages, including form-stability, leakage prevention, and tunable fusion temperature.

**Figure 6:**
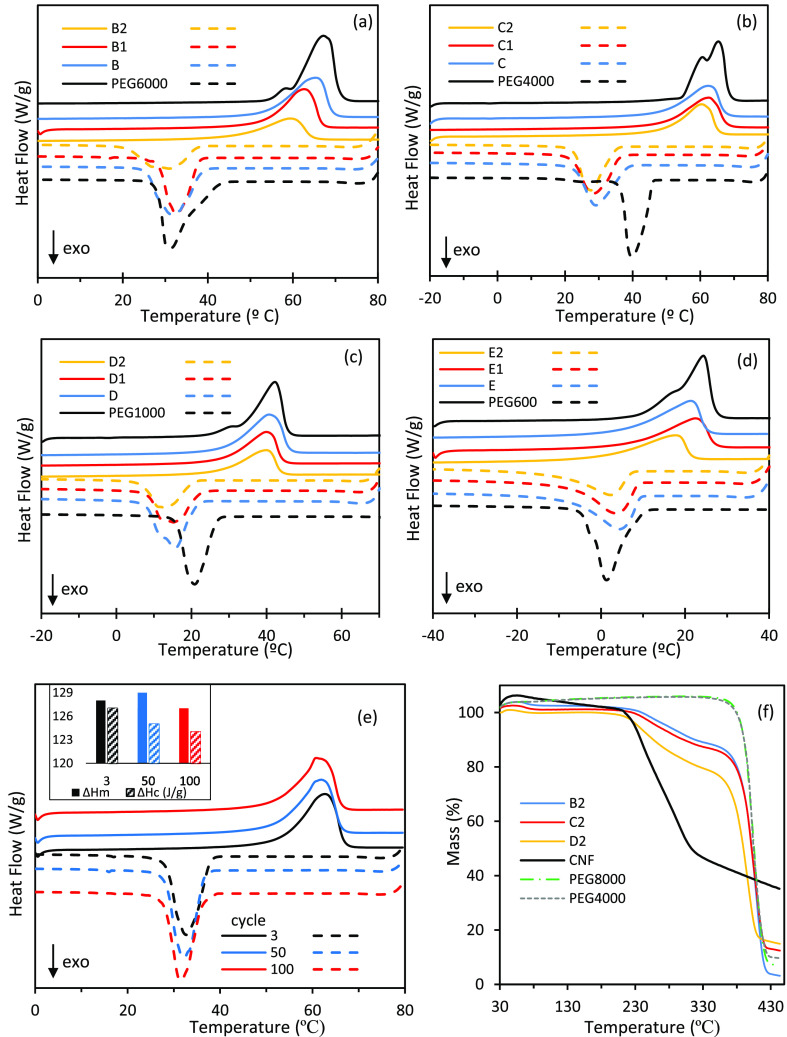
DSC profiles of PCNs following 5 K/min heating–cooling scan rates (lines are heating, and dashes are cooling curves). The compositions include (a) B, PEG6000, (b) C, PEG4000, (c) D, PEG1000, and (d) E, PEG600. See Table 1 for the compositions and Figure S6 for the rest of DSC curves. (e) Repeatability of phase-change behavior of PCN (B1) measured at a 5 K/min DSC scan rate for 100 cycles. (f) TGA profiles of PCN (B2, C2, and D2), CNF, and PEG.

PEG can exist in different forms within the PCN structure.^3^ Free PEG fractions show similar thermal behavior, that is, fusion temperature on the DSC curve (Figure 6), as neat PEG. Since the analyzed PCN compositions showed lowered fusion temperatures and broadened DSC peaks (Figure 6 and Table 1), there is indication that they did not include free PEG. Moderately adsorbed PEG molecules on the CNF can undergo phase transition (freezing-bound, FB), whereas strongly adsorbed PEG molecules are unable to undergo phase transition (non-freezing bound, NFB). The NFB fraction (*W*_NFB_) can be estimated by the following equation^54^
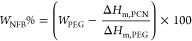
2where *W*_PEG_ is the mass fraction of PEG in the PCN and Δ*H*_m,PCN_ and Δ*H*_m,PEG_ are the enthalpies of melting for the PCN and pure PEG, respectively. The NFB fractions in the PCN compositions correlate with the total mass fraction of CNF and the molecular size of PEG. The fraction of NFB generally increases with increasing PEG molecular mass and higher CNF content in the PCN. By raising the CNF content (15–25 wt %), the W_NFB_ percentage is in the range of 15–24 wt % (PEG8000), 9–25% (PEG6000), 8–22% (PEG4000), 8–17% (PEG1000), and 10–13% (PEG600). Indeed, the higher CNF content and the larger PEG molecular mass can lead to stronger entanglement and intermolecular interactions between these two components in the PCN, as schematically illustrated in Figure 5. This results in a higher NFB fraction in the amorphous–crystal interface and a reduced dimension of the spherulitic lamellae, as shown in Figure 2. The NFB fractions resulting from the adsorption of PEG on CNF in the PCN are consistent with previously reported NFB values of sugar alcohol and water adsorbed within polymeric matrices.^3,55,56^

As presented in Table 1, PCN shows a high loading of PEG (85%) and a high fusion enthalpy, up to 146 J/g, compared with the previously reported polymer-supported PEG, Table 2. For example, up to 60 and 70% of PEG4000 were previously loaded with cellulose and agarose matrices with no melt leakage for an enthalpy of fusion of 84.6 and 110.8 J/g, respectively.^40^ Our PCN provides tailorable phase-changing properties by using the PEG’s molecular weight. The nonleaking retention of the PEG melt is due to its high affinity with the CNF matrices. The PCN demonstrated a remarkable repeatability for thermal cycling, as shown with the over 100 DSC heating-cooling cycles, Figure 6e. The TGA profiles of PCN, CNF, and PEG are illustrated in Figure 6f, confirming the durability of the nanohybrid within the operational temperature range, below 100 °C. CNF and PEG showed a one-step degradation within 215–320 and 350–420 °C temperatures, respectively. A one-stage thermal decomposition within 200–320 °C was previously reported for TEMPO-oxidized CNFs.^57,58^ The degradation profile of PCN results from the combination of CNF and PEG profiles. The degradation of PCN compositions starts at 215 °C and increases with the PEG molecular weight. The PCN shows a two-stage degradation; the first stage ranges from 215 to 350 °C, associated with the nanocellulose decomposition, and the second stage ranges between 350 and 450 °C, related to PEG depolymerization and formation of carbonaceous residues.

**Table 2:** Fusion Temperature and Latent Heat Storage Properties of Different PEG-Polymer-Based Composites in the Literature

material	*T*_m_ (°C)	Δ*H*_m_ (J/g)	Δ*H*_c_ (J/g)	references
PEG6000(93 wt %)-sodium alginate	59.0	156.8	–150.3	(29)
PEG4000 (60 wt %)-cellulose	58.5	84.6	–78.9	(40)
PEG4000 (70 wt %)-agarose	57.7	110.8	–99.0	(40)
PEG4000 (80 wt %)-chitosan	57.1	152.1	–138.3	(40)
PEG10000 (70 wt %)-cellulose acetate		120.1	–104.4	(18)
PEG8000 (96.5 wt %)-cellulose acetate	60.5	155.3		(17)
PEG8000(70 wt %)-synthetic polymer	61.1	131.9	–127.2	(59)
PEG4000(−)-synthetic polymer	43.8	79.6	–85.7	(53)
PEG4000(−)-synthetic polymer	49.9	98.2	–102.0	(53)
**PEG600-8000 (85 wt %)-CNF**	**17.8 to 65.4**	**99 to 146**	**–90 to −139**	**this work**

**Figure 7:**
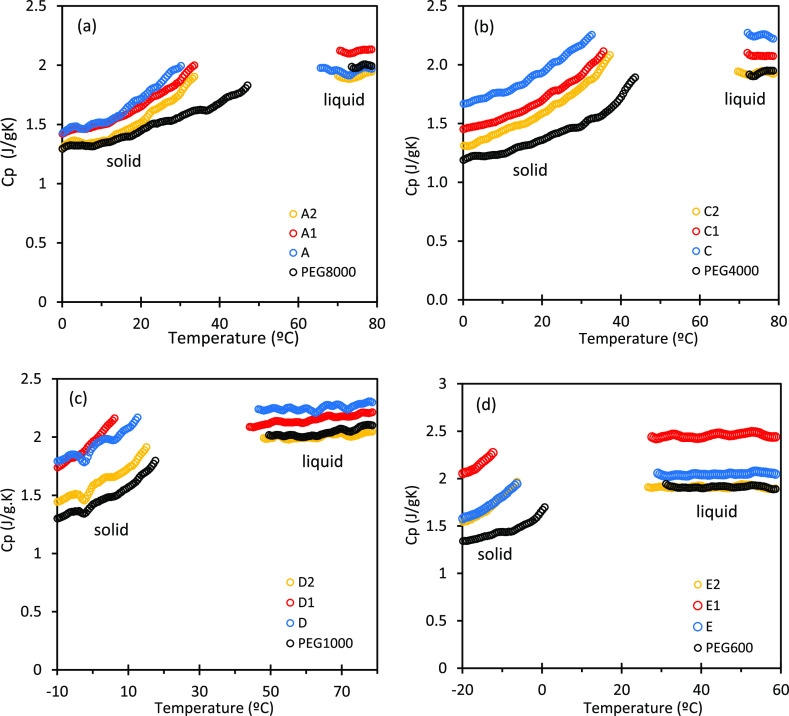
Specific heat capacity (*C*_p_) of the PCN compositions measured by DSC at a 5 K/min heating scan rate: (a) B-compositions for PEG6000, (b) C-compositions for PEG4000, (c) D-compositions for PEG1000, and (d) E-compositions for PEG600. See Table 1 for the compositions and Figure S6 for the rest of *C*_p_ curves. The phase transition regions are not included for simplicity.

Figures 7 and S6 show the specific heat capacity (*C*_p_) of PCNs and starting materials, as measured by DSC. It is observed that the *C*_p_ values of the PCNs are generally higher than those of the PEGs of molecular weights used in this study, both in solid and liquid forms. This increase may be associated with phase transition of the confined PEG within the CNF matrices. In addition, the *C*_p_ of PCN generally increases with an increased mass fraction of the confined PEG, especially in the solid state. In other words, the more the PEG is confined in the CNF matrices, the higher is the PCN’s specific heat capacity. Similar behavior was previously reported for *n*-tetradecane encapsulated in polystyrene–silica.^60^ During heating, PCM is first charged with the sensible heat of the solid up to the fusion onset, when it shifts to the latent heat of fusion. After melting, a further temperature increase is stored as sensible heat of the liquid. The total heat stored (*Q*_t_) in the PCM can be estimated as follows
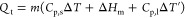
3where *m* is the mass (g) and *C*_p,s_ and *C*_p,l_ are the average specific heat capacities in the Δ*T* and Δ*T*′ temperature ranges for the solid and liquid states, respectively. The *C*_p_ values of the solid and liquid at given temperatures are compiled in Table 1. For instance, the total energy stored by the PCN (B), as sensible and latent heat, when heated within 20–80 °C, is estimated as *Q*_t_ = 194.4 J/g. Table 2 compares the percentage of PEG loading and the latent heat storage properties of different systems including PEG4000 (60 wt %)-cellulose (Δ*H*_m_ = 84.6 J/g),^40^ PEG8000 (96.5 wt %)-cellulose acetate (Δ*H*_m_ = 155.3 J/g),^17^ and PEG4000-synthetic polymer (Δ*H*_m_ = 98.2 J/g).^53^ Note that the specific heat capacity and adjustability of the phase-change properties for these composites have not been considered previously.

### 3.4 Thermal Protection of PCN

**Figure 8:**
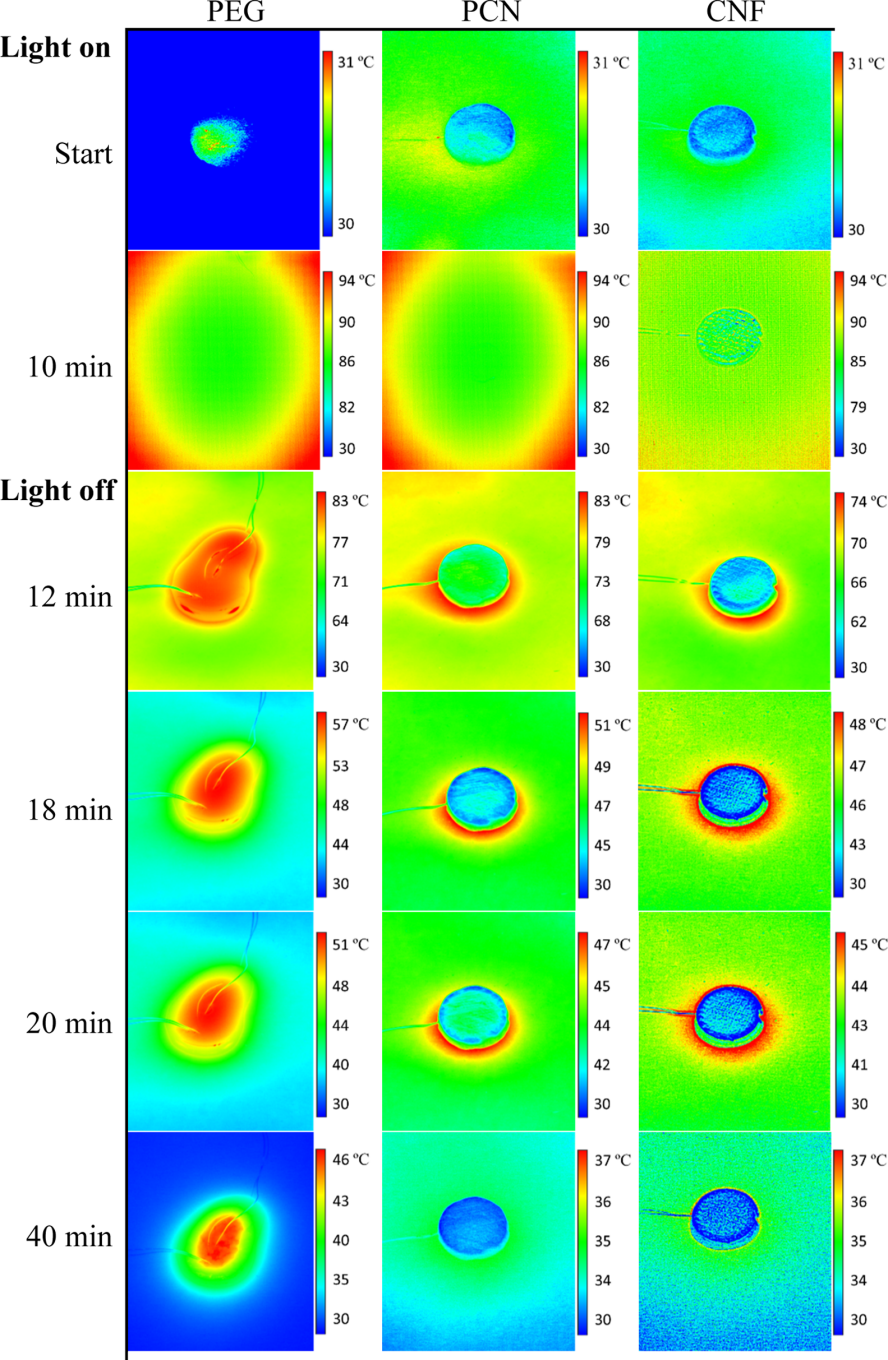
Infrared thermal images of PEG, PCN, and CNF under 10 min light irradiation and 30 min of consecutive cooling and light-off. Note that to keep the surface area of the samples similar under irradiation, the mass of PEG was higher than those of PCN or CNF.

**Figure 9:**
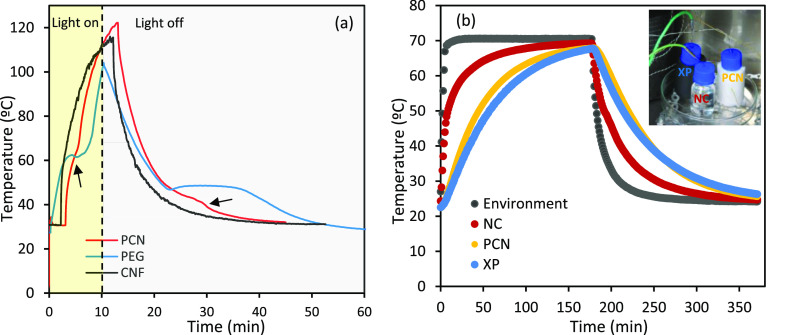
Thermal regulation and light-to-heat conversion performance studied by the thermal camera and thermal chamber. (a) The temperature inside the samples was measured by thermocouples as a function of time. (b) The temperature of water inside the bottles covered with PCN is compared with that of Armaflex insulation material (marked as XP in the inset image) and with no coverage (NC) collected by thermocouples from the thermal chamber set-up. Note that the difference in temperature stabilization during phase change (marked with arrows), for PEG and CNF, stems from differences in the sample mass.

A set of experiments were conducted by using an infrared thermal camera, for example, to visualize the thermal regulation and light-to-heat conversion of the PCN, CNF, and PEG samples. The temperature distribution on the sample surface was recorded by the camera images illustrated in Figure 8. The Supporting Information includes infrared videos of the measurements. The form-stability of PCN against the fluidity of PEG by melting, under light, is clearly visualized in the supplemented videos and Figure 8. The fluid retention of CNF facilitates handling of the PCM melt above the fusion point. The variation of temperature versus time is plotted in Figure 9a, which was measured by a thermocouple inserted in the sample to overcome the emissivity. During the irradiation, the PCN showed a lower temperature than PEG and CNF. By removing the irradiation, the PCN maintained a temperature longer than both the CNF and PEG, until about 45 °C, when heat was released upon crystallization in PCN and PEG. Because of the higher mass of PEG, melting at around 60 °C captured more energy, and consequently, crystallization at around 45 °C released more energy than the respective values for PCN, which caused a more profound temperature stabilization during phase change by PEG compared to that of PCN (Figure 9a). A similar thermal regulation property was reported previously for a rubber-encapsulated PCM.^7^ The total thermal energy stored as sensible and latent heats by the PCN (C2), CNF, and PEG samples was determined using eq 3 (see Figure 7 and Table 1 for the latent heat and C_p_ values measured by DSC) as 206, 130, and 275 J/g, respectively. This study indicates that the addition of CNF improves the fluidity retention, insulation, and TES properties of PEG.

Thermal protection of the PCN was tested with a bottle, which was covered with a 10 mm PCN layer, as shown in Figure S2b. The temperature changes of water inside the bottle are plotted against time in Figure 9b. As can be seen, the PCN insulator outperformed the commercial material as far as the temperature regulation of the liquid inside the bottle. This amounts to about 5 °C during heating and 3.4 °C during cooling. Considering the studied temperature range (22–70 °C), these values suggest up to 10% improvement. This indicates that the PCN can provide reliable protection against thermal shocks or transients, that is, those resulting from sudden changes in the surrounding temperature. The high thermal insulation properties of the PCN and CNF are also confirmed by thermal conductivity measurements. The thermal conductivity values of CNF and PCN (3D-printed C2 composition) are 0.035 and 0.040 W m^–1^ K^–1^, respectively. CNF appears to dominate the conductivity. Porosity is among the most influential criteria for insulation. A high porosity can result in lower thermal conductivity of materials at environmentally relevant temperatures.^61^ Cellulose is used for thermal insulation, for example, in electrical transformers and buildings.^62,63^ Nanocellulose foams and aerogels with high thermal insulation properties were reported previously, within 0.020 and 0.040 W m^–1^ K^–1^ range.^62,64,65^ To put the results into context, lightweight thermal protection materials with low thermal conductivity were investigated in Mars’ Aeroflyby mission, given that vehicle entry in the Martian atmosphere is exposed to significant aerodynamic heating.^35^ Moreover, lightweight structures in buildings provide low thermal inertia and fail to dissipate the heat during hot summers, necessitating the integration of additional energy-storage materials, for example, to increase building thermal inertia.^36^ Therefore, our lightweight biohybrids are promising alternatives for fossil-based insulators in ecofriendly and energy-efficient applications. This is owing to the low conductivity of the CNF and the high thermal inertia of the PCM.

## 4 Conclusions

We demonstrated a lightweight, form-stable PCN that was developed from PEG PCM and CNF. They are introduced for thermal protection and energy-storage applications. As a solid-to-liquid PCM, the use of PEG for temperature-dependent applications is limited because of its fluidity and leakage in the melt state. Herein, the cellulosic nanofibrillar matrices were used as an organogelator for form-stabilization of PEG that prevent these issues. The PCN was prepared via a simple and facile aqueous blending of CNF suspension with PEG, which further enabled processing of the material via different methods, including additive manufacturing, casting/molding, and wet spinning. The enhanced physical interactions between the coupled components in the PCN were revealed by FTIR, DSC, and DMA. The morphology and crystallinity of the developed nanohybrid were characterized by SEM, POM, and XRD, confirming spherulitic crystalline PEG structures within the cellulosic nanofibrillar network. A high loading of PEG (85 wt %) in the PCN was achieved without any leakage in the melt state. This was due to the high miscibility and compatibility of PEG and CNF. The PCN provided a tunable fusion temperature, within 18–65 °C, with PEG of a given molecular mass (600–8000 g/mol range) and latent heat storage of up to 146 J/g. The cycling repeatability of PCN for the latent heat storage was confirmed over 100 DSC heating-cooling cycles. The thermal regulation performance of PCN was demonstrated by an infrared thermal camera under simulated sunlight and thermal chamber tests. The introduced green, lightweight nanohybrid is proposed for TES and management in applications requiring specific working temperatures, smart-energy buildings, aerospace equipment, and waste heat-generating electronics.

## Supporting Information Available

Comparative rheological and viscometry studies of PEG-CNF hydrogels; moduli (*G*′ and *G*″) versus shear stress and angular frequency of PEG-CNF hydrogels; FTIR data of PCN and initial materials in a wide spectral range; schematic of the experimental setup for infrared thermal camera measurement; image of a bottle covered with PCN for thermal regulation and insulation experiments; images of CNF in different forms; form-stability of 3D-printed PCN versus fluidity of PEG under 2 h of heat exposure at 80 °C; and DSC and *C*_p_ curves of different PCN compositions and CNF (PDF)
Crystallization of PEG (MP4)
Crystallization of PCN (MP4)
Infrared camera movies of PEG (MP4)
Infrared camera movies of PCN (MP4)
