# Supplementary material for: Cellulose Nanofibrils Endow Phase-Change Polyethylene Glycol with Form Control and Solid-to-gel Transition for Thermal Energy Storage
Source: ACS Appl Mater Interfaces. 2021 Feb 1;13(5):6188–200. doi: 10.1021/acsami.0c18623 (PMC7944486; doi:10.1021/acsami.0c18623)
Supplement: Supplementary file 1 — am0c18623_si_001.pdf [file am0c18623_si_001.pdf]

Supporting information:

Cellulose nanofibrils endow phase change polyethylene glycol with form control and solid-to-gel transition for thermal energy storage

Maryam R. Yazdani [a], Rubina Ajdary [b], Ari Kankkunen [a], Orlando J. Rojas [b,c], Ari Seppälä [a]*

[a] Department of Mechanical Engineering, School of Engineering, Aalto University, Espoo 02150, Finland

[b] Department of Bioproducts and Biosystems, School of Chemical Technology, Aalto University, Espoo 02150, Finland

[c] Bioproducts Institute, Departments of Chemical & Biological Engineering, Chemistry, and Wood Science, 2360 East Mall, The University of British Columbia, Vancouver, BC V6T 1Z3, Canada

Correspondence: *Maryam R. Yazdani, Email: roza.yazdani@aalto.fi

ORCID: 0000-0002-7057-7994

List of figure captions:

Figure S1. (a) Flow curves with the apparent shear viscosity as a function of shear rate including an image of 3D printed PEG-CNF hydrogel showing an excellent hold of the 3D structure (the sample after drying is illustrated in Figure 1a in the paper); (b) frequency dependency of storage modulus (G′) and loss modulus (G″) for the bulk CNF suspension and hydrogels containing CNF:PEG4000 with the ratios of 60:40, 40:60, 20:80 (wt%). (c) Moduli (G′ and G″) versus shear stress for intermediate PEG-CNF hydrogels; and (d) ATR-FTIR spectra of PEG4000 and PEG8000, PCN (C2 and A), and CNF.

Figure S2. (a) Schematic of experimental set-up for infrared thermal camera measurements. (b) An image of PCN coverage of a bottle for thermal regulation and insulation experiments.

Figure S3. CNF samples in (a) 1D-filament, (b) 2D-film, and (c) 3D-printed forms produced by wet-spinning, casting, and additive manufacturing, respectively.

Figure S4. SEM images of filament cross section (a) CNF and (b) PCN (PEG4000:CNF 75:25 wt%); filament surface (c) CNF and (d) PCN; films (e) CNF and (f) PCN.

Figure S5. Neat PEG4000 and PCN (PEG4000:CNF 75:25 wt%) under 2 hours of heat exposure at 80 ºC demonstrating the retention of PCM melt by the CNF matrix.

Figure S6. (a) DSC curves of PCN A-compositions including PEG8000. (b) C_p of PCN B-compositions including PEG6000. (c) DSC curves of CNF. (d) C_p values of CNF. The measurements were performed under 5 K/min scan rate.

Rheology Measurements. Rheological analysis was conducted to study the behavior of the PEG-CNF hydrogels under processing pressure, because these rheological responses determine

the final structure of the biohybrid. The viscosity of intermediate hydrogels including PEG4000 and CNF with the ratios of 40:60, 60:40, and 80:20 wt% (based on dry mass) was measured by a dynamic rotational rheometer (MCR 302, Anton Paar, Germany) at room temperature. A parallel plate (PP25) with a gap of 1 mm was used to run the test on the hydrogels. The viscosity change was studied within increasing shear rate from 0.01 to 100 s^{-1}. The linear viscoelastic range was quantified by a strain sweep within 0.01 to 100% at a fixed frequency of 10 rad s^{-1}. Frequency sweep was tested using the parallel plate geometry (PP25) with a gap of 0.5 mm and a constant strain of 0.5% over the frequency range of 0.1 to 100 rad s^{-1}. The storage (G') and loss (G") moduli were analyzed within the linear viscoelastic region at a selected constant oscillating strain.

Rheological behavior of PEG-CNF Hydrogel. The viscosity of the intermediate PEG-CNF hydrogels was studied to better understand their gelling behavior under different processing methods. Physically entangled hydrogels were prepared via a facile and low-energy route by adding the given amount of PEG into CNF suspension and mixing at 50 ºC until complete homogenization. Figure S1a includes an image of 3D-printed PEG-CNF hydrogel with a strong hold of the 3D structure, processed by a 3D printer. The rheological behavior of the samples is illustrated in Figure S1. The viscosity of hydrogels decreased with increasing shear rate, which is a shear thinning behavior (Figure S1a). This non-Newtonian behavior is a functional aspect for product processing, especially bioprinting. In such biosystems, the macromolecules can restructure into an aligned arrangement under shear force that results in viscosity reduction during the process. Shear thinning property enables readily dispensing of the fluid system under applied pressure and allows its return to the gel form by time after the stress removal. [1] Increasing concentration of PEG in the hydrogel produced no significant impact on the viscosity, suggesting that PEG does not show adsorption onto nanocellulose in aqueous condition [2] due to the competing effect of water

molecules. Figure S1b presents the plot of G′ and G″ moduli versus oscillatory frequency. The G′>G″ values under the entire frequency sweeps demonstrate a dominant elastic behavior for the hydrogels. The large viscoelastic plateaus of G' and G" moduli along the studied frequency range with no cross point suggest the gel stability. Similar behavior was observed for biopolymer-based hydrogels reported elsewhere. [3, 4] An increasing CNF content in the hydrogels resulted in increasing G′ and G″ moduli. Oscillatory rheology was used to explore the dynamic mechanical behavior of the hydrogel compositions, illustrated in Figure S1c. The G′ values remained higher than those of G″ values for all compositions under a wide shear stress amplitude, which indicates a gel-like behavior. However, exceeding the stress beyond the linear viscoelastic region caused a sharp decrease in G' due to the structural breakdown of the bionetwork. The critical shear stress decreased with increasing PEG content as 24.81, 30.04, and 36.65 Pa for the compositions with PEG/CNF ratios of 80:20, 60:40, and 40:60 wt%, respectively, while the value for bulk CNF was 47.38 Pa. The frequency sweep test was therefore performed at 10 Pa to investigate the stability of various compositions along the entire frequency range of 0.1 to 100 rad/s.

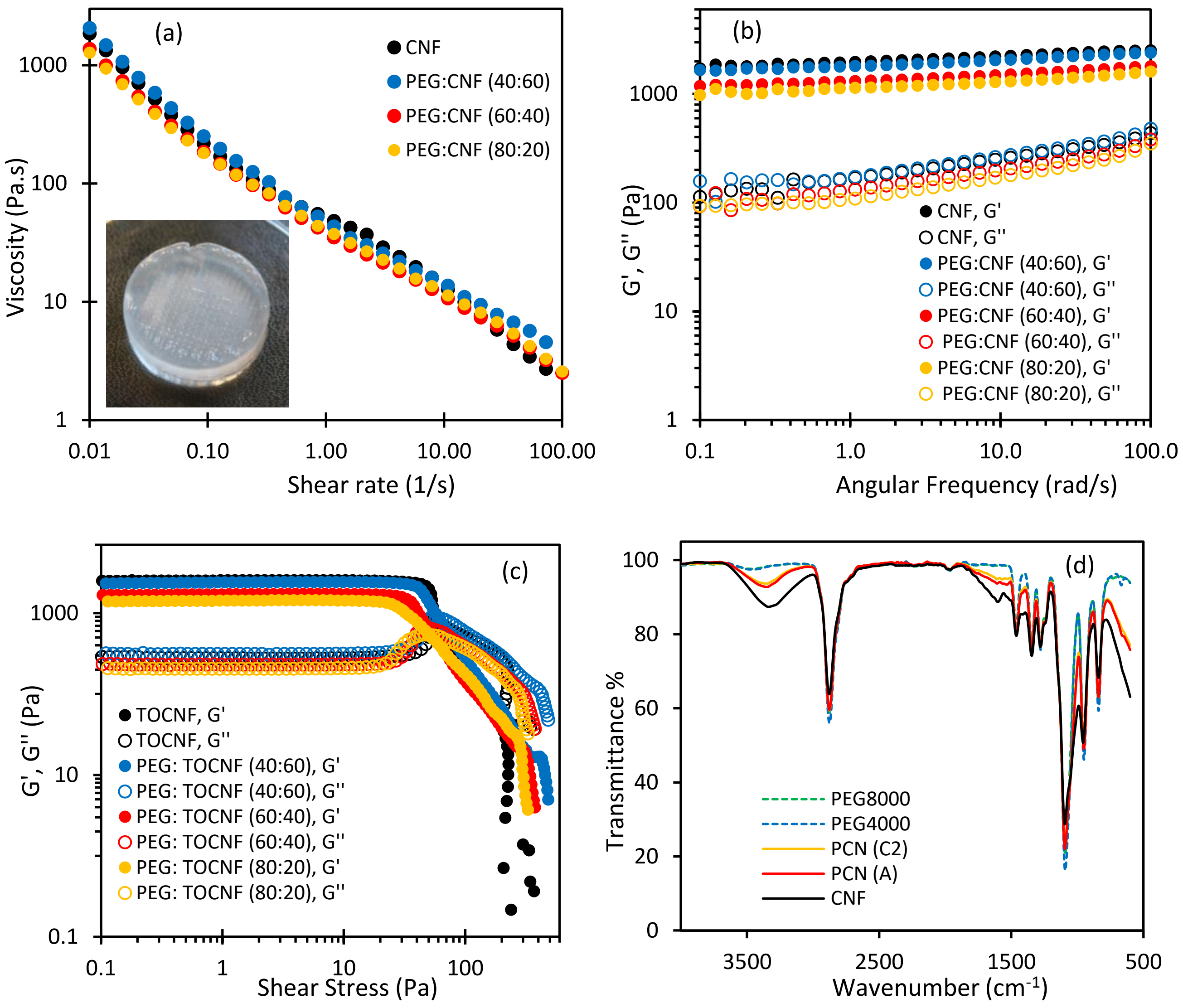

Figure S1. (a) Flow curves with the apparent shear viscosity as a function of shear rate including an image of 3D printed PEG-CNF hydrogel showing an excellent hold of the 3D structure (the sample after drying is illustrated in Figure 1a in the paper); (b) frequency dependency of storage modulus (G′) and loss modulus (G″) for the bulk CNF suspension and hydrogels containing CNF:PEG4000 with the ratios of 60:40, 40:60, 20:80 (wt%). (c) Moduli (G′ and G″) versus shear stress for intermediate PEG-CNF hydrogels; and (d) ATR-FTIR spectra of PEG4000 and PEG8000, PCN (C2 and A), and CNF.

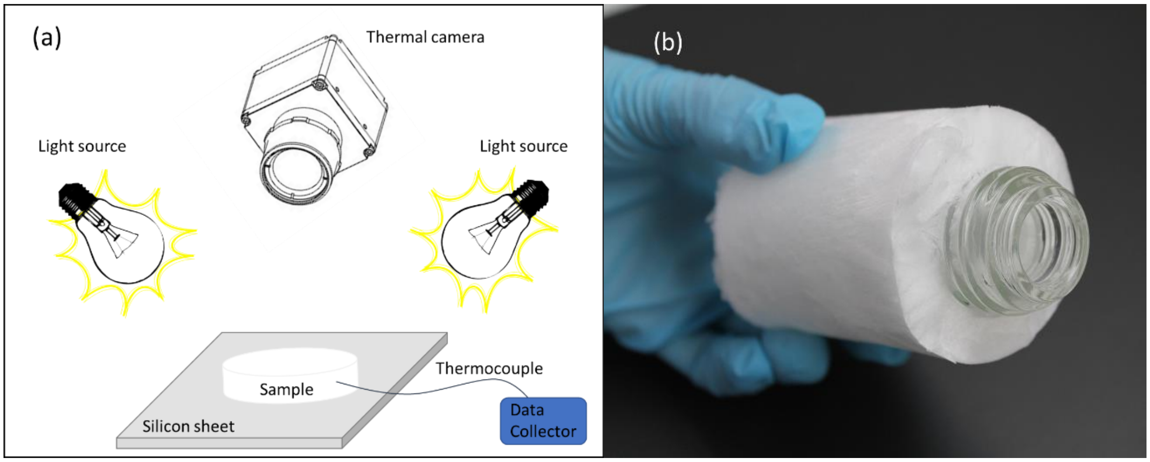

Figure S2. (a) Schematic of experimental set-up for infrared thermal camera measurements. (b) An image of PCN coverage of a bottle for thermal regulation and insulation experiments.

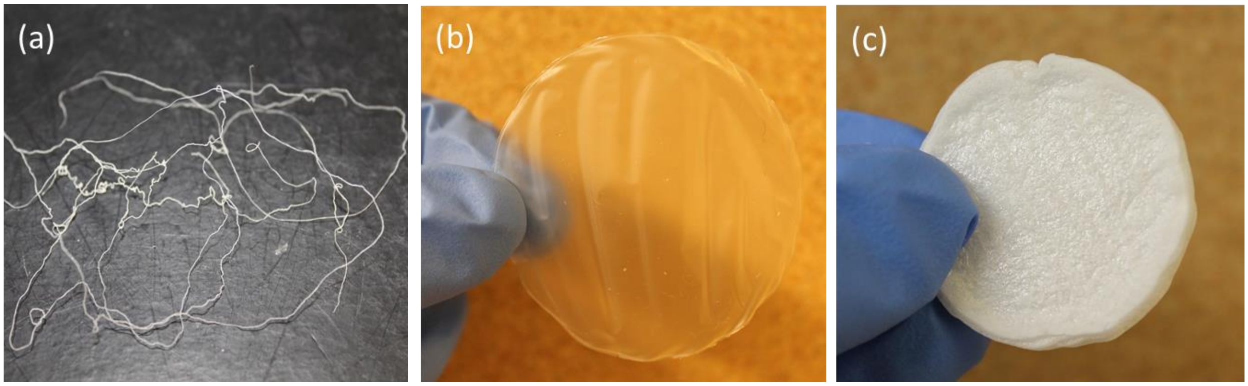

Figure S3. CNF samples in (a) 1D-filament, (b) 2D-film, and (c) 3D-printed forms produced by wet-spinning, casting, and additive manufacturing, respectively.

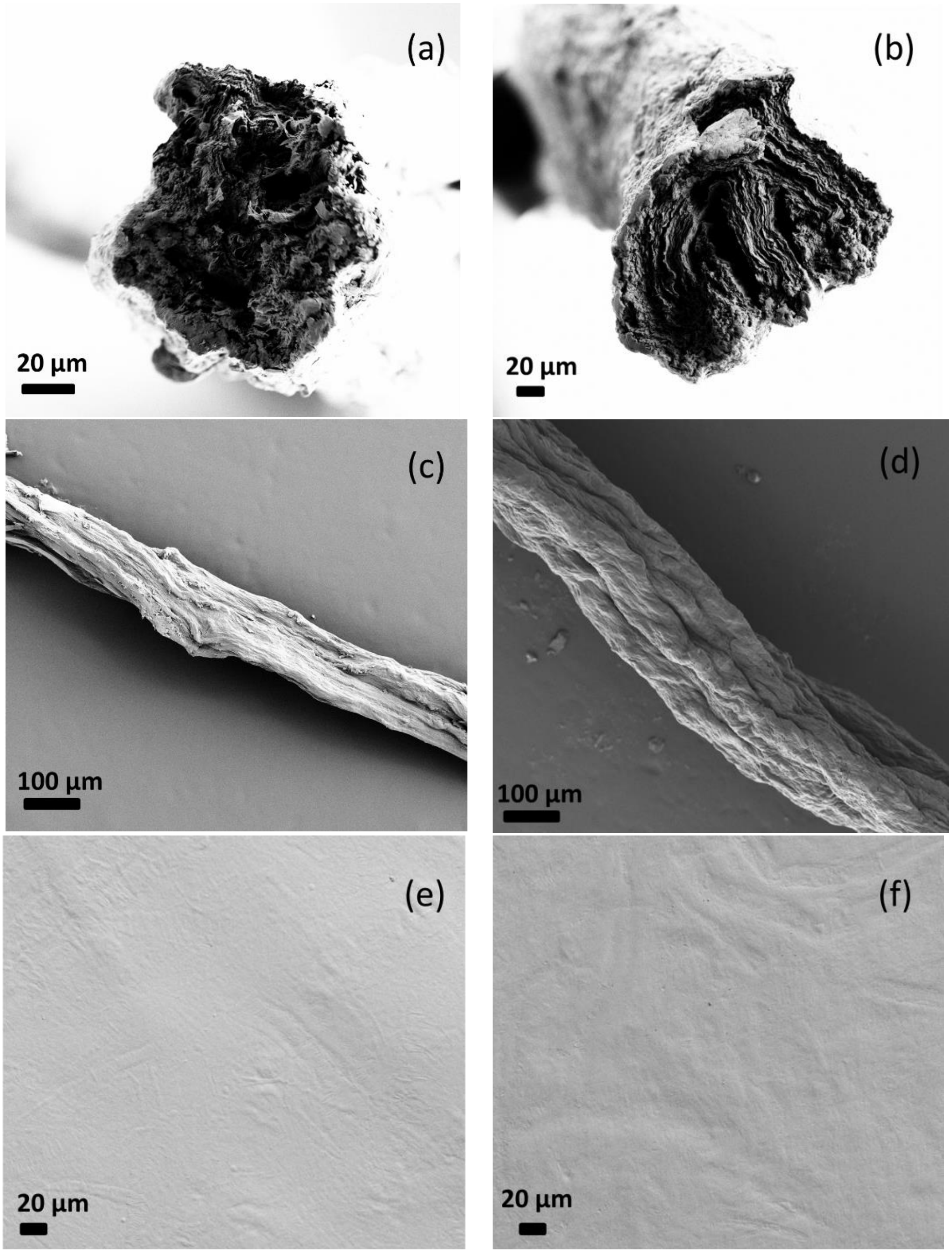

Figure S4. SEM images of filament cross section (a) CNF and (b) PCN (PEG4000:CNF 75:25 wt%); filament surface (c) CNF and (d) PCN; films (e) CNF and (f) PCN.

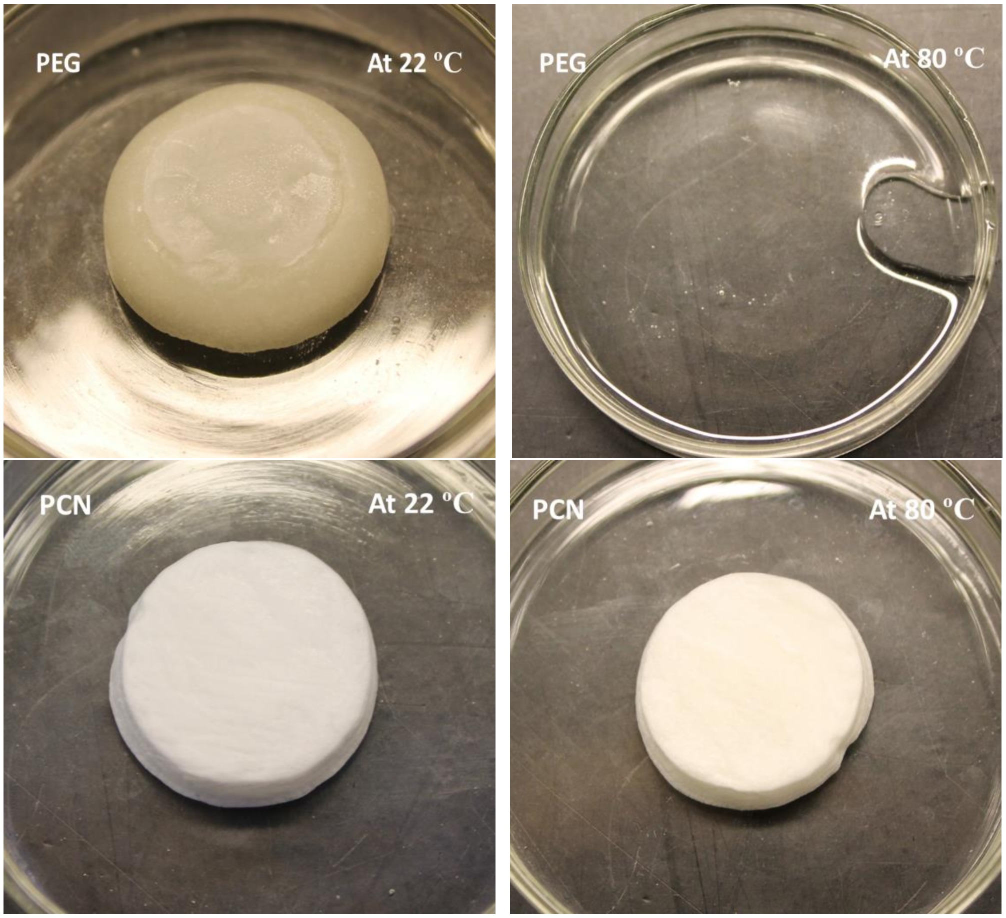

Figure S5. Neat PEG4000 and PCN (PEG4000:CNF 75:25 wt%) under 2 hours of heat exposure at 80 ºC demonstrating the retention of PCM melt by the CNF matrix.

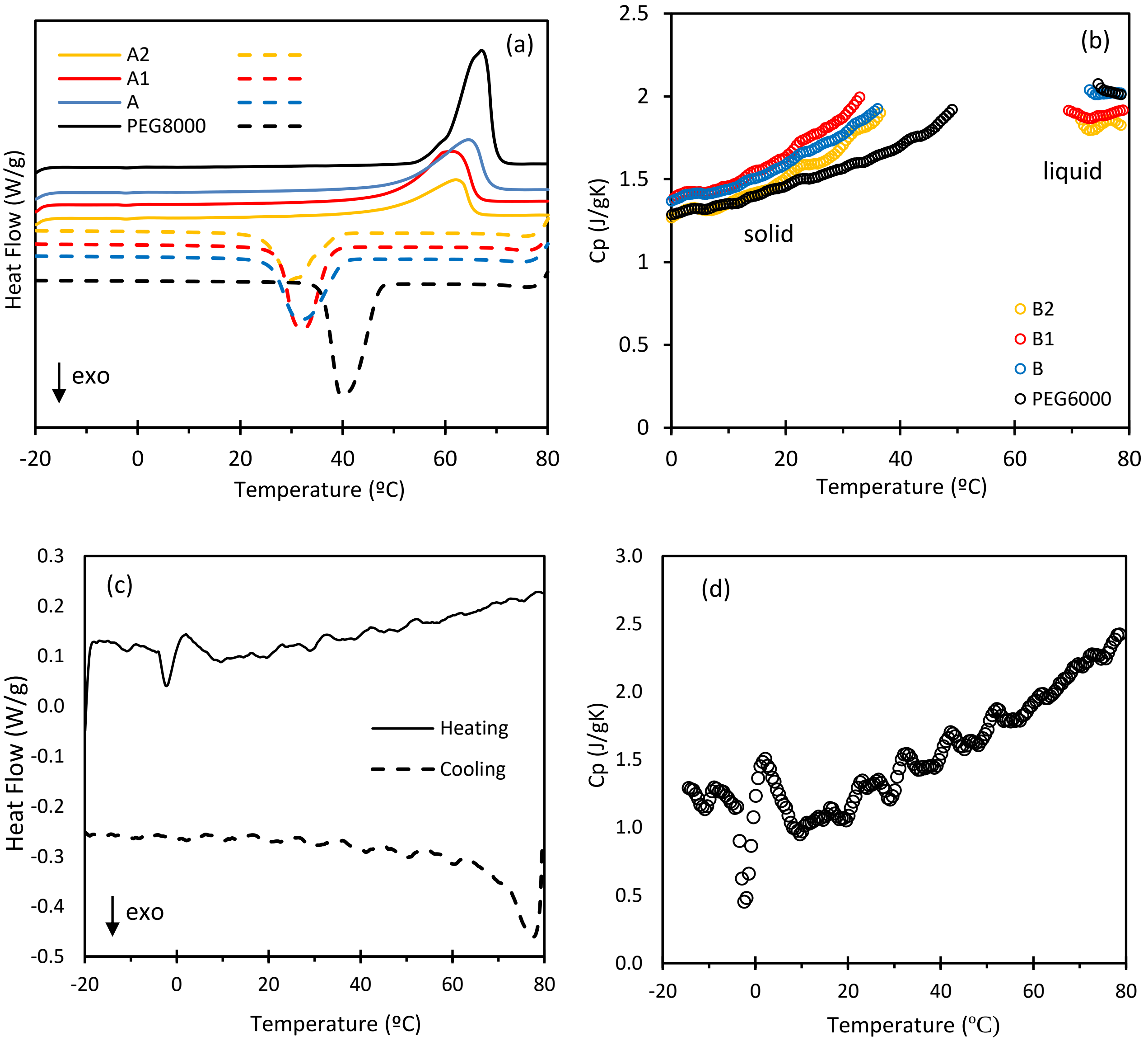

Figure S6. (a) DSC curves of PCN A-compositions including PEG8000. (b) C_p of PCN B-compositions including PEG6000. (c) DSC curves of CNF. (d) C_p values of CNF. The measurements were performed under 5 K/min scan rate.

REFERENCES

1. Min, L. J.; Edgar, T. Y. S.; Zicheng, Z.; Yee, Y. W., Chapter 6 - Biomaterials for Bioprinting. In *3D Bioprinting and Nanotechnology in Tissue Engineering and Regenerative Medicine*, Zhang, L. G.; Fisher, J. P.; Leong, K. W. (Eds.), Academic Press: **2015**; pp 129-148.

2. Reid, M. S.; Marway, H. S.; Moran-Hidalgo, C.; Villalobos, M.; Cranston, E. D., Comparison of polyethylene glycol adsorption to nanocellulose versus fumed silica in water. *Cellulose* **2017,** *24* (11), 4743-4757.

3. Moura, M. J.; Figueiredo, M. M.; Gil, M. H., Rheological Study of Genipin Cross-Linked Chitosan Hydrogels. *Biomacromolecules* **2007,** *8* (12), 3823-3829.

4. Feng, Z.; Simeone, A.; Odelius, K.; Hakkarainen, M., Biobased Nanographene Oxide Creates Stronger Chitosan Hydrogels with Improved Adsorption Capacity for Trace Pharmaceuticals. *ACS Sustain. Chem. Eng.* **2017,** *5* (12), 11525-11535.
